# Supplementary material for: Single-cell proteomics reveals changes in expression during hair-cell development
Source: eLife. 2019 Nov 4;8:e50777. doi: 10.7554/eLife.50777 (PMC6855842; doi:10.7554/eLife.50777)
Supplement: Reporting standard 1. [file elife-50777-repstand1.doc]

**Reporting guidelines for mass spectrometry**

1. General features

1.1 Global descriptors

– Responsible person (or institutional role if more appropriate); provide name, affiliation and stable contact information: **Dr. Ying Zhu, Pacific Northwest National Laboratory, ying.zhu@pnnl.gov, 509-375-4523**

– Instrument manufacturer and model: **ThermoFisher Orbitrap Fusion Lumos Tribrid Mass Spectrometer**

– Customizations (summary): **none**

2. Ion sources

*As each spectrum is acquired using only one ionization source, select the one that applies*

2.1 Electrospray Ionization (ESI)

– Supply type (static, or fed): **fed**

– Interface manufacturer, model: **Thermo Scientific Nanospray Flex™ Ion Source**

– Sprayer type, manufacturer, model: **New Objective** **Self-Pack PicoFrit LC column (i.d. 30 µm) with an integrated electrospray emitter (10 µm tip size)**

– Other parameters if discriminant for the experiment: **LC column packed with 3-µm C18 packing material (300-Å pore size, Phenomenex)**

3. Post-source component

*As an MS spectrum or chromatogram performed on one instrument cannot be acquired using all existing analyzers and detectors, select the elements that apply*

3.1 Analyzer

– Ion optics, ‘simple’ quadrupole, hexapole, Paul trap, linear trap, magnetic sector, FT-ICR, Orbitrap: name of the analyzer(s): **Quadrupole for isolation of precursor ions, Orbitrap detection for both MS1 and MS2.**

3.2 Activation / dissociation

*The associated acquisition parameters are covered in 4.1*

– Instrument component where the activation / dissociation occurs: **ion-routing multipole**

– Gas type (when used) : **Helium**

– Activation / dissociation type : **HCD**

4. Spectrum and peak list generation and annotation

4.1 Data acquisition

– Software name and version: **Xcalibur version 4.1.5**

– Acquisition parameters: **Follows for each prep (experiment)**

**MS acquisition parameters**

**MS precursor scan resolution (MS1): 120,000**

**MS precursor scan AGC (MS1): 3E6**

**MS precursor scan maximum ion injection time (MS1): 254 ms**

**RF lens: 30%**

**Filter MIPS mode: peptide**

**Filter charge state: 2-7**

**Filter dynamic exclusion duration: 40 s**

**Filter minimum intensity: 8000**

**Filter maximum intensity: 1E+20**

**MS tolerance: ±10 ppm**

**HCD collision energy: 30%**

**MS2 scan resolution: 120,000**

**MS2 scan AGC: 1E5**

**MS2 scan Maximum Ion Injection Time: 502 ms**

**Reporting guidelines for protein and peptide** **identification and characterization software**

1. General features

1.1 Global descriptors

– Date stamp (as YYYY-MM-DD) **n/a**

– Responsible person (or institutional role if more appropriate); provide name, affiliation and stable contact information **Peter Barr-Gillespie, Oregon Hearing Research Center, gillespp@ohsu.edu, 503-494-2936**

– Software name, version and manufacturer **MaxQuant 1.5.1.2**

– Customizations made to that software **n/a**

– Availability of that software **maxquant.org**

– Location of the files generated; parameter files, spectral data (input/output) **n/a**

2. Input data and parameters

2.1 Input data

– Description and type of MS data **Thermo .RAW**

– Availability of MS data (source of data, file format) **ProteomeXchange; .RAW files**

2.2 Input parameters

– Databases queried; description and versions (including number of entries searched **xx**

– Taxonomical restrictions applied **n/a**

– Description of tool and scoring scheme **MaxQuant 1.5.1.2**

– Specified cleavage agent(s) **Trypsin/P**

– Allowed number of missed cleavages **2**

– Additional parameters related to cleavage **n/a**

– Permissible amino acids modifications **Acetyl (protein N-term); oxidation (M)**

– Precursor-ion and fragment ion mass tolerance for tandem MS (when applicable) **n/a**

– Mass tolerance for PMF (when applicable) **n/a**

– Thresholds; minimum scores for peptides, proteins (probabilities, number of hits, other metrics) **n/a**

– Any other relevant parameters **n/a**

3. The output from the procedure

*The procedure might generate all or part of the elements described below (identified proteins, identified peptides, quantization information). Select the elements that apply.*

3.1 For identified proteins

– Accession code in the queried database **See data files**

– Protein description **See data files**

– Protein scores **See data files**

– Validation status **See data files**

– Number of different peptide sequences (without considering modifications) assigned to the protein **See data files**

– Percent peptide coverage of protein **See data files**

– Identity of supporting peptides **See data files**

– In the case of PMF, number of matched/unmatched peaks **n/a**

3.2 For identified peptides

– Sequence (indicate any deviation from the expected protein cleavage specificity) **See data files**

– Peptide scores **See data files**

– Chemical modifications (artefactual) and post-translational modifications (naturally occurring); sequence polymorphisms with experimental evidence (particularly for isobaric modifications) **See data files**

– Corresponding spectrum locus **n/a**

– Charge assumed for identification and a measurement of peptide mass error **See data files**

– Other additional information, when used for evaluation of confidence **See data files**

3.3 Quantitation for selected ions

– Quantitation approach (*e.g.* 4plex-iTRAQ, ICAT, cICAT, COFRADIC) **Relative iBAQ**

– Quantity measurement (*e.g.* integration of signals, use of signal intensity) **Integration of MS1 peak area**

– Data transformation and normalization technique (description of method and software) **MaxQuant 1.5.1.2 and Excel; see below**

– Number of replicates (biological and technical) **3-10 samples (biological replicates) per condition; one injection (technical replicate) per sample**

– Acceptance criteria (including measure of errors) **n/a**

– Estimates of uncertainty and the methods for the error analysis, including the treatment of relevant systematic error effects and the treatment of random error issues **n/a**

– Results from controls (when described) **n/a**

4. Interpretation and validation

– Assessment and confidence given to the identification and quantitation (description of methods, thresholds, values, etc,) **n/a**

– Results of statistical analysis or determination of false positive rate in case of large scale experiments **n/a**

– Inclusion/exclusion of the output of the software are provided (description of what part of the output has been kept, what part has been rejected) **All contaminants rejected; all reversed entries are present in output spreadsheet but are not used in quantitation calculations**

**Reporting guidelines for the peptide and protein quantification analysis.**

***1. General features***

– Experiment identifier or name **n/a**

– Responsible person or role **Peter Barr-Gillespie, Oregon Hearing Research Center, gillespp@ohsu.edu, 503-494-2936**

– Quantitative approach **Relative iBAQ; label-free extracted ion chromatograms, with intensity converted to iBAQ and iBAQ converted to riBAQ (Krey et al., J. Proteome Res. 13, 1034; 2013).**

***2. Experimental design and sample description***

2.1. Experimental design

– Groups **FACS-isolated embryonic day 15 chick utricle cells, labeled with FM1-43; cells were detected as FM-high (likely hair cells) and FM-low (likely supporting cells); cells collected into nanoPOTS nanoliter-scale wells (0, 1, 3, 5, or 20 cells per well)**

– Biological and technical replicates **3-10 samples (biological replicates) per condition; one injection (technical replicate) per sample**

2.2. Sample / Assay description

– Labeling protocol (if applicable) **n/a**

– Sample description **See tables below**

Sample name **See tables below**

Sample amount **See tables below**

Sample labeling with assay definition, i.e. MS run / data set together with reporting ion mass, reagent or isotope labeled amino acid **n/a**

Replicates and/or groups **n/a**

Isotopic correction coefficients **n/a**

**Internal references n/a**

| **Experiment 1** |  |  |  |  |  |
| --- | --- | --- | --- | --- | --- |
| Analysis 2018-11-19a |  |  |  |  |  |
| MaxQuant with Match Between Runs |  |  |  |  |  |
| Gal_gal_v77.Galgal4.pep.all.fixed.extras.fasta | |  |  |  |  |
|  |  |  |  |  |  |
|  |  |  |  |  |  |
| **Sample name** | **FM1-43 signal** | **Cells per well** | **Biological replicate** | **Technical replicate** | **RAW file name** |
| 700526_20cell_High_090618_1 | High | 20 | 1 | 1 | 700526_Single_Hair_Cell_OHSU_20cell_High_090618_1_30um_031218_300bar.raw |
| 700533_20cell_High_090618_2 | High | 20 | 2 | 1 | 700533_Single_Hair_Cell_OHSU_20cell_High_090618_2_30um_031218_300bar.raw |
| 700539_20cell_High_090618_3 | High | 20 | 3 | 1 | 700539_Single_Hair_Cell_OHSU_20cell_High_090618_3_30um_031218_300bar.raw |
| 700567_20cell_High_090618_4 | High | 20 | 4 | 1 | 700567_Single_Hair_Cell_OHSU_20cell_High_090618_4_30um_031218_300bar.raw |
| 700576_5cell_High_090618_1 | High | 5 | 1 | 1 | 700576_Single_Hair_Cell_OHSU_5cell_High_090618_1_30um_031218_300bar.raw |
| 700579_5cell_High_090618_2 | High | 5 | 2 | 1 | 700579_Single_Hair_Cell_OHSU_5cell_High_090618_2_30um_031218_300bar.raw |
| 700584_5cell_High_090618_3 | High | 5 | 3 | 1 | 700584_Single_Hair_Cell_OHSU_5cell_High_090618_3_30um_031218_300bar.raw |
| 700730_5cell_High_090618_4 | High | 5 | 4 | 1 | 700730_Single_Hair_Cell_OHSU_5cell_High_090618_4_30um_031218_300bar.raw |
| 700739_3cell_High_090618_1 | High | 3 | 1 | 1 | 700739_Single_Hair_Cell_OHSU_3cell_High_090618_1_30um_031218_300bar.raw |
| 700740_3cell_High_090618_2 | High | 3 | 2 | 1 | 700740_Single_Hair_Cell_OHSU_3cell_High_090618_2_30um_031218_300bar.raw |
| 700750_3cell_High_090618_3 | High | 3 | 3 | 1 | 700750_Single_Hair_Cell_OHSU_3cell_High_090618_3_30um_031218_300bar.raw |
| 701129_3cell_High_090618_4 | High | 3 | 4 | 1 | 701129_Single_Hair_Cell_OHSU_3cell_High_090618_4_30um_031218_300bar.raw |
| 701155_1cell_High_090618_1 | High | 1 | 1 | 1 | 701155_Single_Hair_Cell_OHSU_1cell_High_090618_1_30um_031218_300bar.raw |
| 701157_1cell_High_090618_2 | High | 1 | 2 | 1 | 701157_Single_Hair_Cell_OHSU_1cell_High_090618_2_30um_031218_300bar.raw |
| 701166_1cell_High_090618_3 | High | 1 | 3 | 1 | 701166_Single_Hair_Cell_OHSU_1cell_High_090618_3_30um_031218_300bar.raw |
| 701176_1cell_High_090618_4 | High | 1 | 4 | 1 | 701176_Single_Hair_Cell_OHSU_1cell_High_090618_4_30um_031218_300bar.raw |
| 701190_1cell_High_090618_5 | High | 1 | 5 | 1 | 701190_Single_Hair_Cell_OHSU_1cell_High_090618_5_30um_031218_300bar.raw |
| 701199_20cell_Low_090618_2 | Low | 20 | 1 | 1 | 701199_Single_Hair_Cell_OHSU_20cell_Low_090618_2_30um_031218_300bar.raw |
| 701229_20cell_Low_090618_3 | Low | 20 | 2 | 1 | 701229_Single_Hair_Cell_OHSU_20cell_Low_090618_3_30um_031218_300bar.raw |
| 701234_20cell_Low_090618_4 | Low | 20 | 3 | 1 | 701234_Single_Hair_Cell_OHSU_20cell_Low_090618_4_30um_031218_300bar.raw |
| 701241_20cell_Low_090618_5 | Low | 20 | 4 | 1 | 701241_Single_Hair_Cell_OHSU_20cell_Low_090618_5_30um_031218_300bar.raw |
| 701243_5cell_Low_090618_1 | Low | 5 | 1 | 1 | 701243_Single_Hair_Cell_OHSU_5cell_Low_090618_1_30um_031218_300bar.raw |
| 701250_5cell_Low_090618_3 | Low | 5 | 2 | 1 | 701250_Single_Hair_Cell_OHSU_5cell_Low_090618_3_30um_031218_300bar.raw |
| 701251_5cell_Low_090618_2 | Low | 5 | 3 | 1 | 701251_Single_Hair_Cell_OHSU_5cell_Low_090618_2_30um_031218_300bar.raw |
| 701253_5cell_Low_090618_4 | Low | 5 | 4 | 1 | 701253_Single_Hair_Cell_OHSU_5cell_Low_090618_4_30um_031218_300bar.raw |
| 701278_3cell_Low_090618_1 | Low | 3 | 1 | 1 | 701278_Single_Hair_Cell_OHSU_3cell_Low_090618_1_30um_031218_300bar.raw |
| 701280_3cell_Low_090618_2 | Low | 3 | 2 | 1 | 701280_Single_Hair_Cell_OHSU_3cell_Low_090618_2_30um_031218_300bar.raw |
| 701282_3cell_Low_090618_3 | Low | 3 | 3 | 1 | 701282_Single_Hair_Cell_OHSU_3cell_Low_090618_3_30um_031218_300bar.raw |
| 701295_1cell_Low_090618_1 | Low | 1 | 1 | 1 | 701295_Single_Hair_Cell_OHSU_1cell_Low_090618_1_30um_031218_300bar.raw |
| 701368_1cell_Low_090618_2 | Low | 1 | 2 | 1 | 701368_Single_Hair_Cell_OHSU_1cell_Low_090618_2_30um_031218_300bar.raw |
| 701372_1cell_Low_090618_3 | Low | 1 | 3 | 1 | 701372_Single_Hair_Cell_OHSU_1cell_Low_090618_3_30um_031218_300bar.raw |
| 701376_1cell_Low_090618_4 | Low | 1 | 4 | 1 | 701376_Single_Hair_Cell_OHSU_1cell_Low_090618_4_30um_031218_300bar.raw |
| 701377_1cell_Low_090618_5 | Low | 1 | 5 | 1 | 701377_Single_Hair_Cell_OHSU_1cell_Low_090618_5_30um_031218_300bar.raw |

| **Experiment 2** |  |  |  |  |  |
| --- | --- | --- | --- | --- | --- |
| Analysis 2019-04-22b |  |  |  |  |  |
| MaxQuant with Match Between Runs | |  |  |  |  |
| Gal_gal_v77.Galgal4.pep.all.fixed.extras.fasta | | |  |  |  |
|  |  |  |  |  |  |
|  |  |  |  |  |  |
| **Sample name** | **FM1-43 signal** | **Cells per well** | **Biological replicate** | **Technical replicate** | **RAW file name** |
| rHigh_1_R01 | High | 1 | 1 | 1 | Single_Hair_Cell_OHSU_1cell_High_030819_R1_YF30um_350bar.raw |
| rHigh_1_R02 | High | 1 | 2 | 1 | Single_Hair_Cell_OHSU_1cell_High_030819_R2_YF30um_350bar.raw |
| rHigh_1_R03 | High | 1 | 3 | 1 | Single_Hair_Cell_OHSU_1cell_High_030819_R3_YF30um_350bar.raw |
| rHigh_1_R04 | High | 1 | 4 | 1 | Single_Hair_Cell_OHSU_1cell_High_030819_R4_YF30um_350bar.raw |
| rHigh_1_R05 | High | 1 | 5 | 1 | Single_Hair_Cell_OHSU_1cell_High_030819_R5_YF30um_350bar.raw |
| rHigh_1_R06 | High | 1 | 6 | 1 | Single_Hair_Cell_OHSU_1cell_High_030819_R6_YF30um_350bar.raw |
| rHigh_1_R07 | High | 1 | 7 | 1 | Single_Hair_Cell_OHSU_1cell_High_030819_R7_YF30um_350bar.raw |
| rHigh_1_R08 | High | 1 | 8 | 1 | Single_Hair_Cell_OHSU_1cell_High_030819_R8_YF30um_350bar.raw |
| rHigh_1_R09 | High | 1 | 9 | 1 | Single_Hair_Cell_OHSU_1cell_High_030819_R9_YF30um_350bar.raw |
| rHigh_1_R10 | High | 1 | 10 | 1 | Single_Hair_Cell_OHSU_1cell_High_030819_R10_YF30um_350bar.raw |
| rHigh_20_R01 | High | 20 | 1 | 1 | Single_Hair_Cell_OHSU_20cell_High_030819_R1_YF30um_350bar.raw |
| rHigh_20_R02 | High | 20 | 2 | 1 | Single_Hair_Cell_OHSU_20cell_High_030819_R2_YF30um_350bar.raw |
| rHigh_20_R03 | High | 20 | 3 | 1 | Single_Hair_Cell_OHSU_20cell_High_030819_R3_YF30um_350bar.raw |
| rLow_1_R01 | High | 1 | 1 | 1 | Single_Hair_Cell_OHSU_1cell_Low_030819_R1_YF30um_350bar.raw |
| rLow_1_R02 | High | 1 | 2 | 1 | Single_Hair_Cell_OHSU_1cell_Low_030819_R2_YF30um_350bar.raw |
| rLow_1_R03 | High | 1 | 3 | 1 | Single_Hair_Cell_OHSU_1cell_Low_030819_R3_YF30um_350bar.raw |
| rLow_1_R04 | High | 1 | 4 | 1 | Single_Hair_Cell_OHSU_1cell_Low_030819_R4_YF30um_350bar.raw |
| rLow_1_R05 | Low | 1 | 5 | 1 | Single_Hair_Cell_OHSU_1cell_Low_030819_R5_YF30um_350bar.raw |
| rLow_1_R06 | Low | 1 | 6 | 1 | Single_Hair_Cell_OHSU_1cell_Low_030819_R6_YF30um_350bar.raw |
| rLow_1_R07 | Low | 1 | 7 | 1 | Single_Hair_Cell_OHSU_1cell_Low_030819_R7_YF30um_350bar.raw |
| rLow_1_R08 | Low | 1 | 8 | 1 | Single_Hair_Cell_OHSU_1cell_Low_030819_R8_YF30um_350bar.raw |
| rLow_1_R09 | Low | 1 | 9 | 1 | Single_Hair_Cell_OHSU_1cell_Low_030819_R9_YF30um_350bar.raw |
| rLow_1_R10 | Low | 1 | 10 | 1 | Single_Hair_Cell_OHSU_1cell_Low_030819_R10_YF30um_350bar.raw |
| rLow_20_R01 | Low | 20 | 1 | 1 | Single_Hair_Cell_OHSU_20cell_Low_030819_R1_YF30um_350bar.raw |
| rLow_20_R02 | Low | 20 | 2 | 1 | Single_Hair_Cell_OHSU_20cell_Low_030819_R2_YF30um_350bar.raw |
| rLow_20_R03 | Low | 20 | 3 | 1 | Single_Hair_Cell_OHSU_20cell_Low_030819_R3_YF30um_350bar.raw |
| rNoise_R01 | Low | 0 | 1 | 1 | Single_Hair_Cell_OHSU_NoiseControl_030819_R1_YF30um_350bar.raw |
| rNoise_R02 | Low | 0 | 2 | 1 | Single_Hair_Cell_OHSU_NoiseControl_030819_R2_YF30um_350bar.raw |
| rNoise_R03 | Low | 0 | 3 | 1 | Single_Hair_Cell_OHSU_NoiseControl_030819_R3_YF30um_350bar.raw |

| **Experiment 1 + 2** |  |  |  |  |  |
| --- | --- | --- | --- | --- | --- |
| Analysis 2019-05-09a |  |  |  |  |  |
| MaxQuant with Match Between Runs | | |  |  |  |
| v94_Chicken_Gallus_gallus_GRCg6a_pep_all_fixed_additions_no_ex.fasta | | | | |  |
|  |  |  |  |  |  |
|  |  |  |  |  |  |
| **Sample name** | **FM1-43 signal** | **Cells per well** | **Biological replicate** | **Technical replicate** | **RAW file name** |
| 1H1a | High | 20 | 1 | 1 | 700526_Single_Hair_Cell_OHSU_20cell_High_090618_1_30um_031218_300bar.raw |
| 1H1b | High | 20 | 2 | 1 | 700533_Single_Hair_Cell_OHSU_20cell_High_090618_2_30um_031218_300bar.raw |
| 1H1c | High | 20 | 3 | 1 | 700539_Single_Hair_Cell_OHSU_20cell_High_090618_3_30um_031218_300bar.raw |
| 1H1d | High | 20 | 4 | 1 | 700567_Single_Hair_Cell_OHSU_20cell_High_090618_4_30um_031218_300bar.raw |
| 1H1e | High | 5 | 1 | 1 | 700576_Single_Hair_Cell_OHSU_5cell_High_090618_1_30um_031218_300bar.raw |
| 1H20a | High | 5 | 2 | 1 | 700579_Single_Hair_Cell_OHSU_5cell_High_090618_2_30um_031218_300bar.raw |
| 1H20b | High | 5 | 3 | 1 | 700584_Single_Hair_Cell_OHSU_5cell_High_090618_3_30um_031218_300bar.raw |
| 1H20c | High | 5 | 4 | 1 | 700730_Single_Hair_Cell_OHSU_5cell_High_090618_4_30um_031218_300bar.raw |
| 1H20d | High | 3 | 1 | 1 | 700739_Single_Hair_Cell_OHSU_3cell_High_090618_1_30um_031218_300bar.raw |
| 1H3a | High | 3 | 2 | 1 | 700740_Single_Hair_Cell_OHSU_3cell_High_090618_2_30um_031218_300bar.raw |
| 1H3b | High | 3 | 3 | 1 | 700750_Single_Hair_Cell_OHSU_3cell_High_090618_3_30um_031218_300bar.raw |
| 1H3c | High | 3 | 4 | 1 | 701129_Single_Hair_Cell_OHSU_3cell_High_090618_4_30um_031218_300bar.raw |
| 1H3d | High | 1 | 1 | 1 | 701155_Single_Hair_Cell_OHSU_1cell_High_090618_1_30um_031218_300bar.raw |
| 1H5a | High | 1 | 2 | 1 | 701157_Single_Hair_Cell_OHSU_1cell_High_090618_2_30um_031218_300bar.raw |
| 1H5b | High | 1 | 3 | 1 | 701166_Single_Hair_Cell_OHSU_1cell_High_090618_3_30um_031218_300bar.raw |
| 1H5c | High | 1 | 4 | 1 | 701176_Single_Hair_Cell_OHSU_1cell_High_090618_4_30um_031218_300bar.raw |
| 1H5d | High | 1 | 5 | 1 | 701190_Single_Hair_Cell_OHSU_1cell_High_090618_5_30um_031218_300bar.raw |
| 1L1a | Low | 20 | 1 | 1 | 701199_Single_Hair_Cell_OHSU_20cell_Low_090618_2_30um_031218_300bar.raw |
| 1L1b | Low | 20 | 2 | 1 | 701229_Single_Hair_Cell_OHSU_20cell_Low_090618_3_30um_031218_300bar.raw |
| 1L1c | Low | 20 | 3 | 1 | 701234_Single_Hair_Cell_OHSU_20cell_Low_090618_4_30um_031218_300bar.raw |
| 1L1d | Low | 20 | 4 | 1 | 701241_Single_Hair_Cell_OHSU_20cell_Low_090618_5_30um_031218_300bar.raw |
| 1L1e | Low | 5 | 1 | 1 | 701243_Single_Hair_Cell_OHSU_5cell_Low_090618_1_30um_031218_300bar.raw |
| 1L20a | Low | 5 | 2 | 1 | 701250_Single_Hair_Cell_OHSU_5cell_Low_090618_3_30um_031218_300bar.raw |
| 1L20b | Low | 5 | 3 | 1 | 701251_Single_Hair_Cell_OHSU_5cell_Low_090618_2_30um_031218_300bar.raw |
| 1L20c | Low | 5 | 4 | 1 | 701253_Single_Hair_Cell_OHSU_5cell_Low_090618_4_30um_031218_300bar.raw |
| 1L20d | Low | 3 | 1 | 1 | 701278_Single_Hair_Cell_OHSU_3cell_Low_090618_1_30um_031218_300bar.raw |
| 1L3a | Low | 3 | 2 | 1 | 701280_Single_Hair_Cell_OHSU_3cell_Low_090618_2_30um_031218_300bar.raw |
| 1L3b | Low | 3 | 3 | 1 | 701282_Single_Hair_Cell_OHSU_3cell_Low_090618_3_30um_031218_300bar.raw |
| 1L3c | Low | 1 | 1 | 1 | 701295_Single_Hair_Cell_OHSU_1cell_Low_090618_1_30um_031218_300bar.raw |
| 1L5a | Low | 1 | 2 | 1 | 701368_Single_Hair_Cell_OHSU_1cell_Low_090618_2_30um_031218_300bar.raw |
| 1L5b | Low | 1 | 3 | 1 | 701372_Single_Hair_Cell_OHSU_1cell_Low_090618_3_30um_031218_300bar.raw |
| 1L5c | Low | 1 | 4 | 1 | 701376_Single_Hair_Cell_OHSU_1cell_Low_090618_4_30um_031218_300bar.raw |
| 1L5d | Low | 1 | 5 | 1 | 701377_Single_Hair_Cell_OHSU_1cell_Low_090618_5_30um_031218_300bar.raw |
|  |  |  |  |  |  |
| 2H1a | High | 1 | 1 | 1 | Single_Hair_Cell_OHSU_1cell_High_030819_R1_YF30um_350bar.raw |
| 2H1b | High | 1 | 2 | 1 | Single_Hair_Cell_OHSU_1cell_High_030819_R2_YF30um_350bar.raw |
| 2H1c | High | 1 | 3 | 1 | Single_Hair_Cell_OHSU_1cell_High_030819_R3_YF30um_350bar.raw |
| 2H1d | High | 1 | 4 | 1 | Single_Hair_Cell_OHSU_1cell_High_030819_R4_YF30um_350bar.raw |
| 2H1f | High | 1 | 5 | 1 | Single_Hair_Cell_OHSU_1cell_High_030819_R5_YF30um_350bar.raw |
| 2H1g | High | 1 | 6 | 1 | Single_Hair_Cell_OHSU_1cell_High_030819_R6_YF30um_350bar.raw |
| 2H1h | High | 1 | 7 | 1 | Single_Hair_Cell_OHSU_1cell_High_030819_R7_YF30um_350bar.raw |
| 2H1i | High | 1 | 8 | 1 | Single_Hair_Cell_OHSU_1cell_High_030819_R8_YF30um_350bar.raw |
| 2H1j | High | 1 | 9 | 1 | Single_Hair_Cell_OHSU_1cell_High_030819_R9_YF30um_350bar.raw |
| 2H20a | High | 20 | 1 | 1 | Single_Hair_Cell_OHSU_20cell_High_030819_R1_YF30um_350bar.raw |
| 2H20b | High | 20 | 2 | 1 | Single_Hair_Cell_OHSU_20cell_High_030819_R2_YF30um_350bar.raw |
| 2H20c | High | 20 | 3 | 1 | Single_Hair_Cell_OHSU_20cell_High_030819_R3_YF30um_350bar.raw |
| 2L1a | Low | 1 | 1 | 1 | Single_Hair_Cell_OHSU_1cell_Low_030819_R1_YF30um_350bar.raw |
| 2L1b | Low | 1 | 2 | 1 | Single_Hair_Cell_OHSU_1cell_Low_030819_R2_YF30um_350bar.raw |
| 2L1c | Low | 1 | 3 | 1 | Single_Hair_Cell_OHSU_1cell_Low_030819_R3_YF30um_350bar.raw |
| 2L1d | Low | 1 | 4 | 1 | Single_Hair_Cell_OHSU_1cell_Low_030819_R4_YF30um_350bar.raw |
| 2L1f | Low | 1 | 5 | 1 | Single_Hair_Cell_OHSU_1cell_Low_030819_R5_YF30um_350bar.raw |
| 2L1g | Low | 1 | 6 | 1 | Single_Hair_Cell_OHSU_1cell_Low_030819_R6_YF30um_350bar.raw |
| 2L1h | Low | 1 | 7 | 1 | Single_Hair_Cell_OHSU_1cell_Low_030819_R7_YF30um_350bar.raw |
| 2L1i | Low | 1 | 8 | 1 | Single_Hair_Cell_OHSU_1cell_Low_030819_R8_YF30um_350bar.raw |
| 2L1j | Low | 1 | 9 | 1 | Single_Hair_Cell_OHSU_1cell_Low_030819_R9_YF30um_350bar.raw |
| 2L20a | Low | 20 | 1 | 1 | Single_Hair_Cell_OHSU_20cell_Low_030819_R1_YF30um_350bar.raw |
| 2L20b | Low | 20 | 2 | 1 | Single_Hair_Cell_OHSU_20cell_Low_030819_R2_YF30um_350bar.raw |
| 2L20c | Low | 20 | 3 | 1 | Single_Hair_Cell_OHSU_20cell_Low_030819_R3_YF30um_350bar.raw |
| 2N0a | Low | 0 | 1 | 1 | Single_Hair_Cell_OHSU_NoiseControl_030819_R1_YF30um_350bar.raw |
| 2N0b | Low | 0 | 2 | 1 | Single_Hair_Cell_OHSU_NoiseControl_030819_R2_YF30um_350bar.raw |
| 2N0c | Low | 0 | 3 | 1 | Single_Hair_Cell_OHSU_NoiseControl_030819_R3_YF30um_350bar.raw |

***3. Input data***

*Description and reference of the dataset used for quantitative analysis (no actual values).*

– Input data type **Thermo .RAW**

– Input data format **n/a**

– Input data merging **n/a**

– Availability of the input data **ProteomeXchange**

***4. Protocol***

*Description of the software and methods applied in the quantitative analysis (including transformation functions, aggregation functions and statistical calculations).*

4.1. Quantification software name, version and manufacturer **iBAQ calculated with MaxQuant 1.5.1.2; all other calculations done with Microsoft Excel**

4.2. Description of the selection and/or matching method of features, together with the description of the method of the primary extracted quantification values determination for each feature and/or peptide **By MaxQuant with Match Between Runs (default parameters)**

4.3. Confidence filter of features or peptides prior to quantification **By MaxQuant**

4.4. Description of data calculation and transformation methods

– Missing values imputation and outliers removal **n/a**

– Quantification values calculation and / or ratio determination from the primary extracted quantification values **riBAQ for each protein or protein group is iBAQ divided by the sum of all non-contaminant, non-reversed iBAQ values**

– Replicate aggregation **Replicates averaged together**

– Normalization **riBAQ intrinsically normalizes**

– Inference protocol for calculating protein quantification values from peptide quantification values **By MaxQuant**

– Protocol specific corrections **n/a**

4.5. Description of methods for (statistical) estimation of correctness **Reversed database used for FDR estimate at protein level**

4.6. Calibration curves of standards **Assumption is that riBAQ is equal to relative molar abundance (Krey et al., J. Proteome Res. 13, 1034; 2013)**

***5. Resulting data***

*The actual quantification values resulting from your quantification software together with their estimated confidence*

*5.1.* Quantification values at feature and/or at peptide level

– Primary extracted quantification values for each feature, with their statistical estimation of correctness **n/a**

– Quantification values for each peptide as a result of the aggregation of the values of the previous section (5.1.1), with their statistical estimation of correctness **Reversed database used for FDR estimate of 1% at peptide level**

5.2. Quantification values at protein level

– Basic / raw quantification values with statistical estimation of correctness **Reversed database used for FDR estimate of 1% at protein level**

– Transformed / aggregated / combined quantification values of the proteins at group level, with their statistical estimation of correctness **FDR re-evaluated after protein grouping**
